# Supplementary material for: The Remarkable and Selective In Vitro Cytotoxicity of Synthesized Bola-Amphiphilic Nanovesicles on Etoposide-Sensitive and -Resistant Neuroblastoma Cells
Source: Nanomaterials (Basel). 2024 Sep 16;14(18):1505. doi: 10.3390/nano14181505 (PMC11434613; doi:10.3390/nano14181505)
Supplement: Supplementary file 1 [file nanomaterials-14-01505-s001.zip › nanomaterials-3158297-supplementary.pdf]

Supplementary Materials

Section S1. BPPB Characterization

1,1-(1,12-dodecanediyl)bis[1,1,1]-triphenylphosphonium di-Bromide (BPPB)

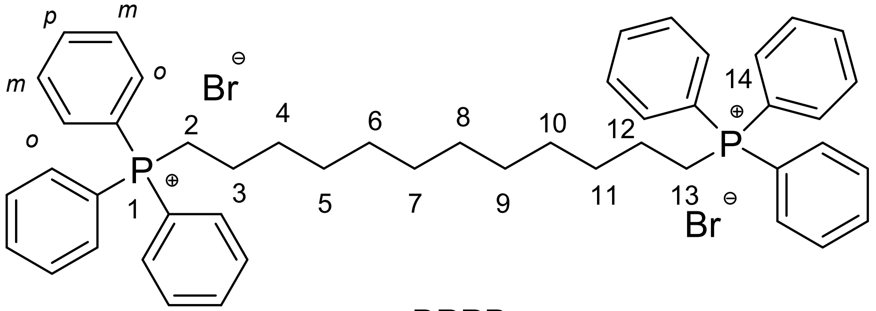

BPPB

Figure S1. Chemical structure of the BPPB with atoms numbering for NMR peaks assignation.

The final product was obtained as hygroscopic low-melting point white foam (2.18 g, 0.0026 moles, 85.2%). M. p. < 70 °C. ATR-FTIR (ν, cm^{-1}): 3052, 3007 (C-H stretching phenyl rings), 2923 (CH_2), 2852 (CH_2), 1586, 1484 (C=C stretching), 1436, 995 (aromatic C-P), 689 (aliphatic C-P). ^{1}H-NMR (400 MHz, $CDCl_3$) ppm: 1.16 (m, 4H, [6,9] CH_2), 1.22 (m, 4H, [5,10] CH_2), 1.23 (m, 4H, [7,8] CH_2), 1.50 (m, 4H, [4,11] CH_2), 1.60 (m, 4H, [3,12] CH_2), 3.62 (dt, 4H, [2,13] CH_2, $J_{CH2(2,13)\text{-}P}$ = 13.0 Hz, J = 6.20 Hz), 7.63 (m, 12H, *meta* ArH), 7.67 (m, 6H, *para* ArH), 7.95 (m, 12H, *orto* ArH). ^{13}C-NMR (100 MHz, $CDCl_3$) ppm: 21.94 (2d, [3,12] C); 22.26 (2d, [2,13] C, $J_{C(2,13)\text{-}P}$ = 50.10 Hz); 28.60 (2s, [7,8] C); 29.39 (2d, [6,9] C); 29.55 (2d, [5,10] C); 30.17 (2d, [4,11] C); 118.32 (6d, ArC-P, $J_{ArC\text{-}P}$ = 86.0 Hz); 130.09 (12d, ArCH*orto*, $J_{ArCHorto\text{-}P}$ = 12.5 Hz); 133.53 (12d, ArCH*meta*, $J_{ArCHmeta\text{-}P}$ = 10.2 Hz); 134.72 (6s, ArCH*para*). ^{31}P-NMR (162 MHz, $CDCl_3$) ppm: 26.72 (s, P nuclei 1,14). FIA-MS-(ESI): 346.25 m/z $[C_{48}H_{54}P_2]^{2+}$. Anal. Calcd. for $C_{48}H_{54}P_2Br_2$: C, 64.61; H, 6.38; P, 7.26. Found: C, 64.63; H, 6.41; P, 7.30.

Table S1. Main characteristics of BPPB.

Analysis	BPPB NPs
ATR-FTIR [cm^{-1}]	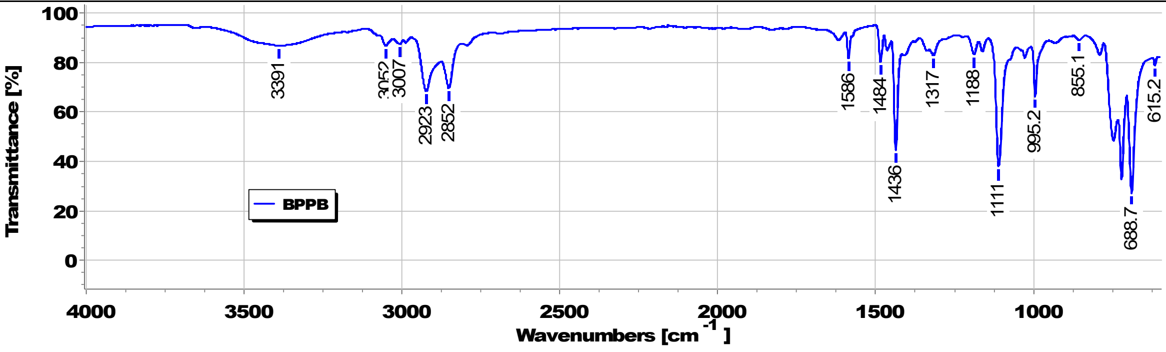

^{1}H NMR (400 MHz, $CDCl_3$)	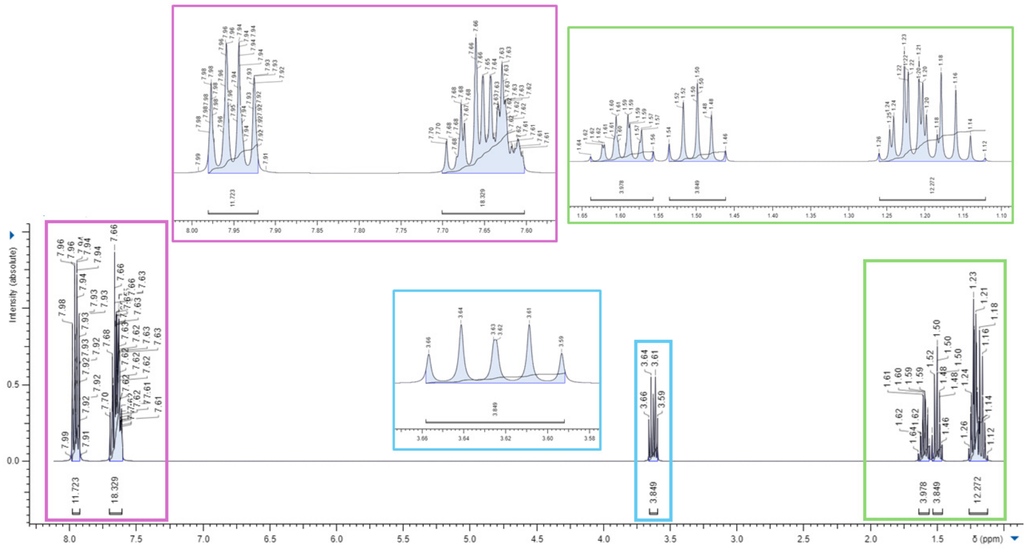
^{13}C NMR (100 MHz, $CDCl_3$)	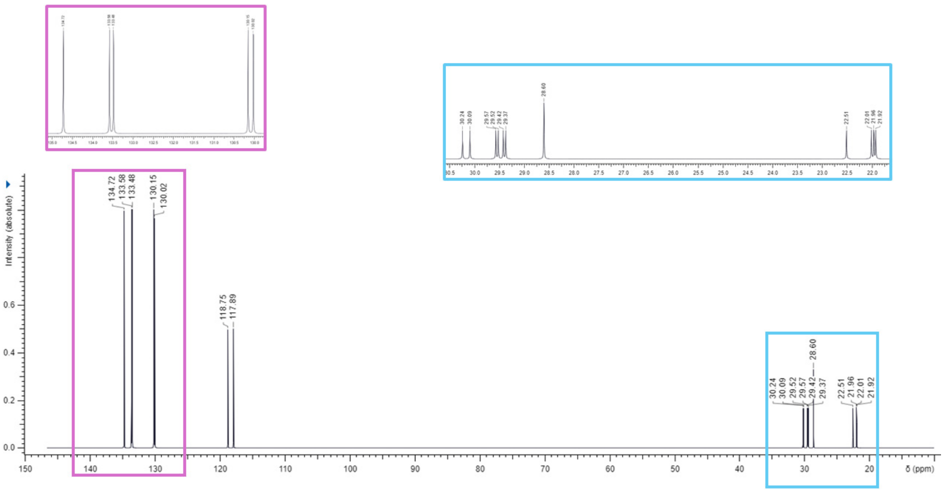
^{13}C NMR DEPT 135 (100 MHz, $CDCl_3$) ^{31}P NMR (red square) (162 MHz, $CDCl_3$)	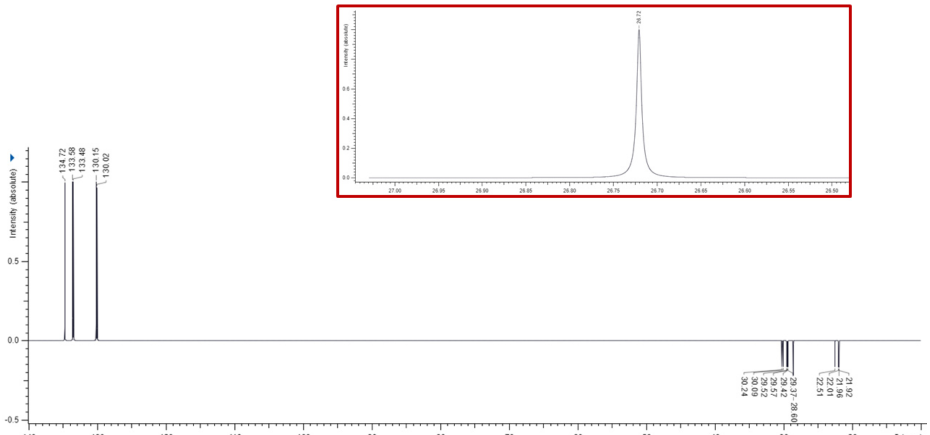

Z-Ave (nm) and PDI (**a**)<br>ζ-p (mV) (**b**)	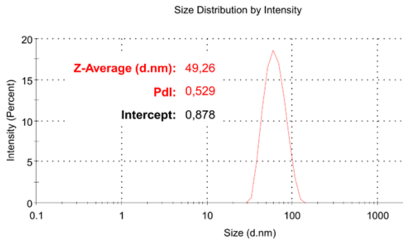 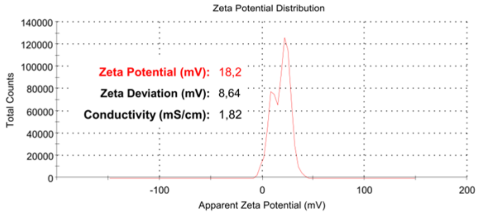 (a) (b)
UV-Vis Spectrum	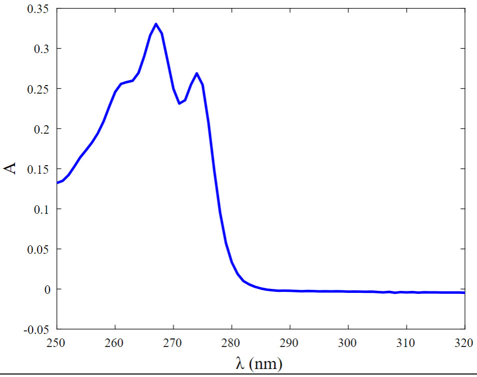
IC_{50} COS-7 cell (μg/mL) *<br>IC_{50} HepG2 cell (μg/mL) *	5.76 ± 0.95<br>11.31 ± 1.54
MICs Gram-positive (μg/mL)<br>MICs Gram-negative (μg/mL)	0.25-0.50<br>1.00-32.00
SIs Gram-positive **<br>SIs Gram-negative **	23-46<br>0.4-11.3
Potentiometric Titration #	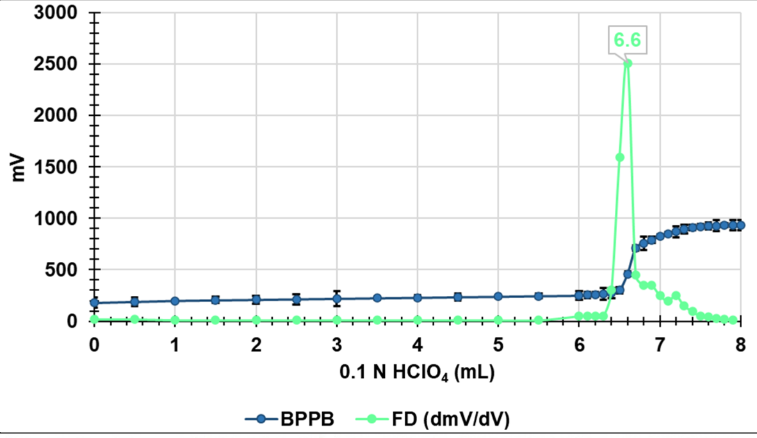
Optical Microscopy<br>Captured with a 40 × objective (**a**)<br>Captured with a 100 × objective (**b**)	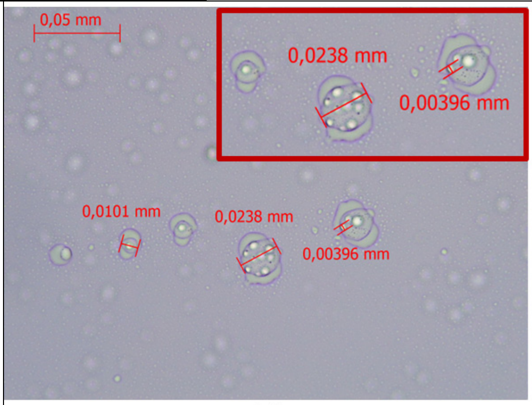 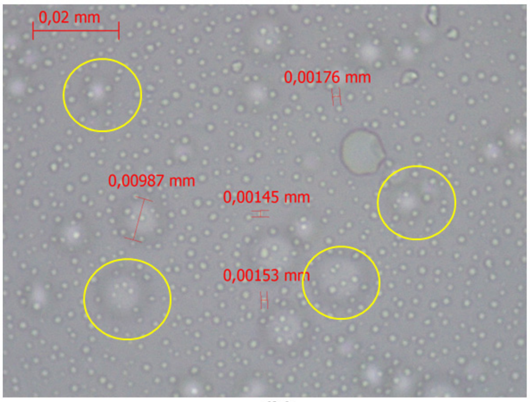 (a) (b)

Z-Ave = hydrodynamic diameter of particle; PDI = polydispersity indices; ζ-p = measure of the electrical charge of particles suspended in the liquid of acquisition (water); # the image also shows the titration curve and the first derivative of BPPB; * MTT test; ** respect to HepG2 cells.

Section S2. Biological Results

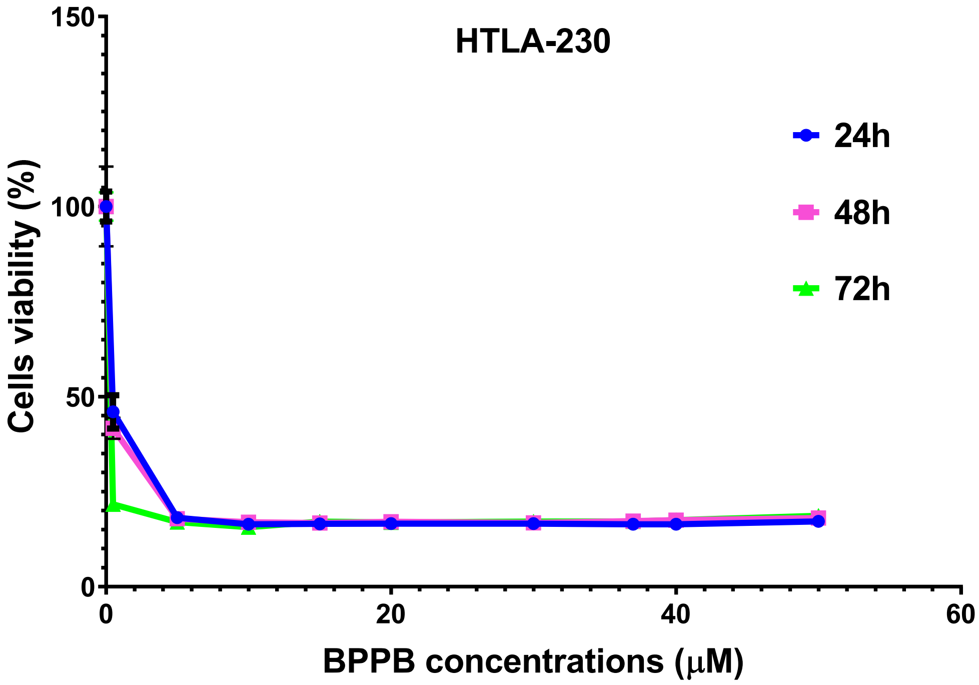

Figure S2. Cell viability (%) of HTLA-230 vs increasing BPPB concentrations (0.5-50 μM) after 24 hours (blue line), 48 hours (pink line) and 72 hours (green line) of exposure. Concentration = 0.0 μM corresponded to the control.

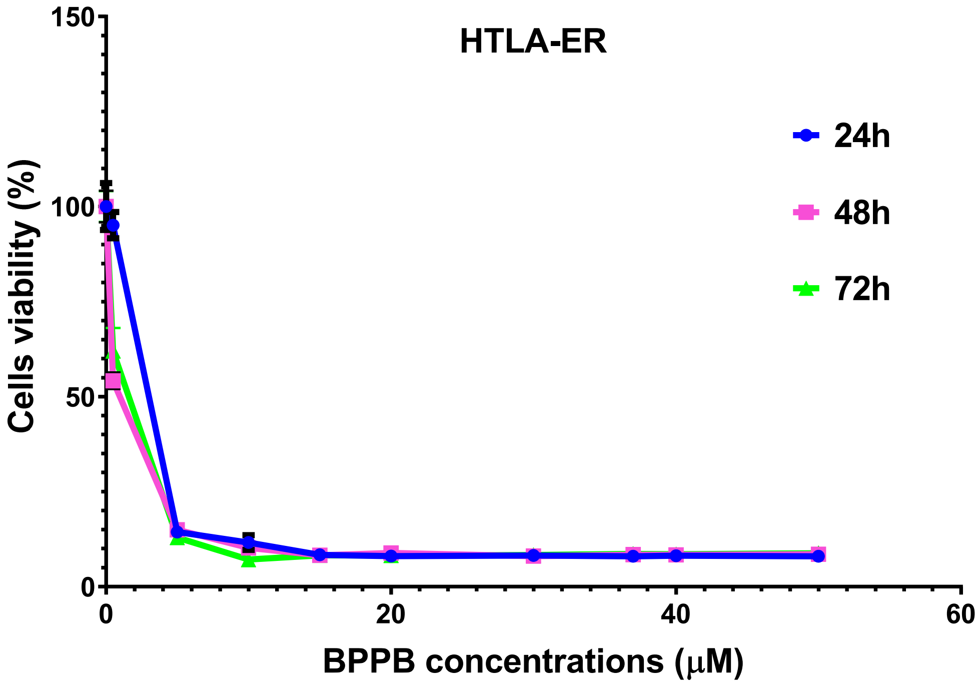

Figure S3. Cell viability (%) of HTLA-ER vs increasing BPPB concentrations (0.5-50 μM) after 24 hours (blue line), 48 hours (pink line) and 72 hours (green line) of exposure. Concentration = 0.0 μM corresponded to the control.

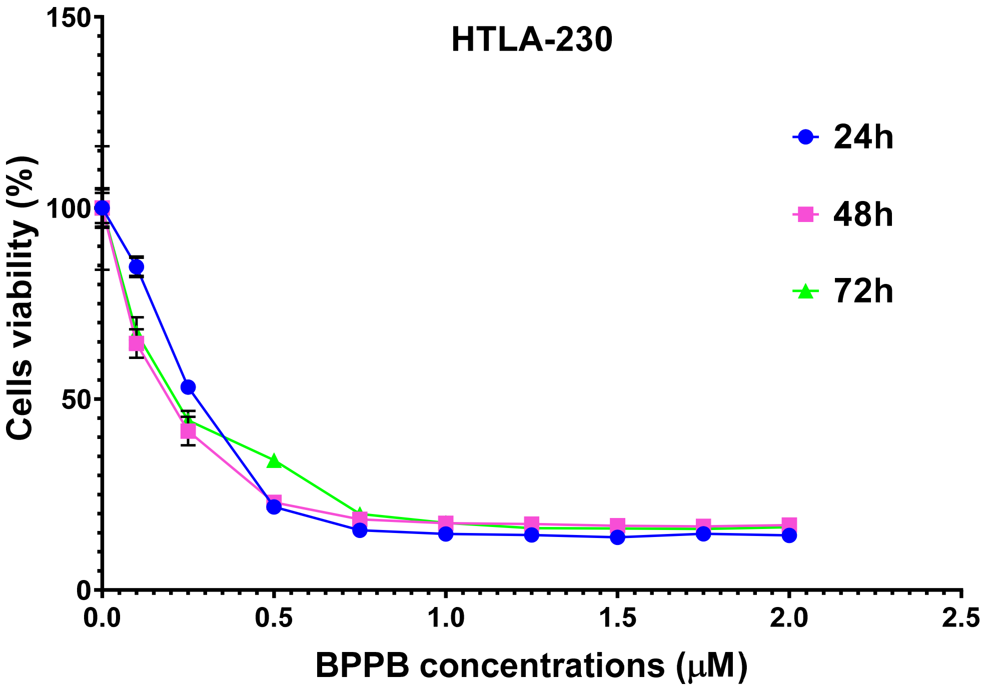

Figure S4. Cell viability (%) of HTLA-230 vs increasing BPPB concentrations (0.1-2 μM) after 24 hours (blue line) 48 hours (pink line) and 72 hours (green line) of exposure. Concentration = 0.0 μM corresponded to the control.

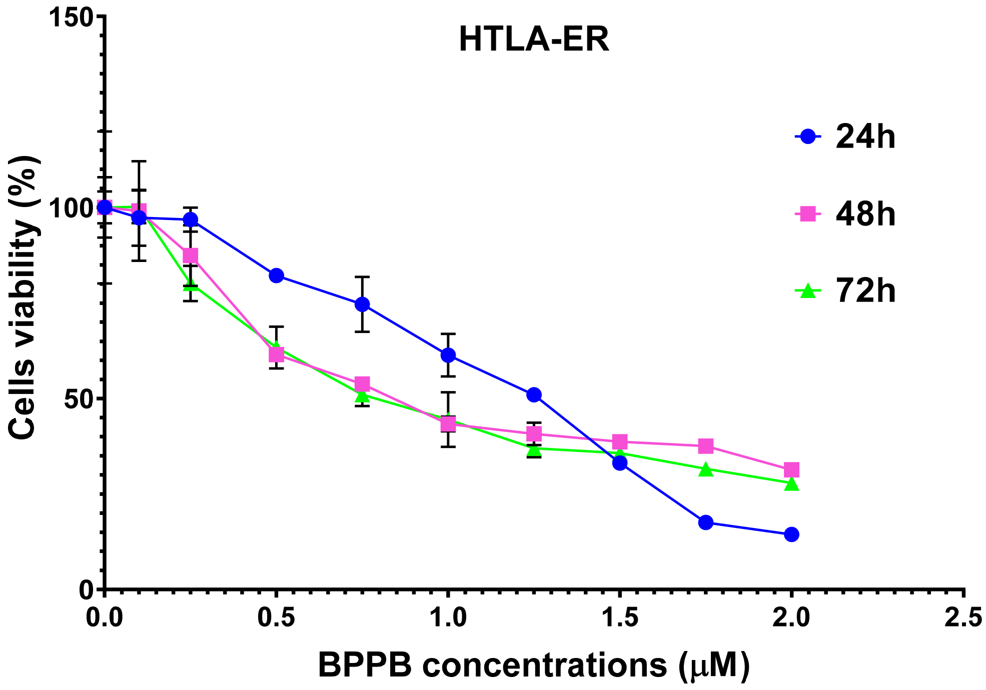

Figure S5. Cell viability (%) of HTLA-ER vs increasing BPPB concentrations (0.1-2 μM) after 24 hours (blue line) 48 hours (pink line) and 72 hours (green line) of exposure. Concentration = 0.0 μM corresponded to the control.

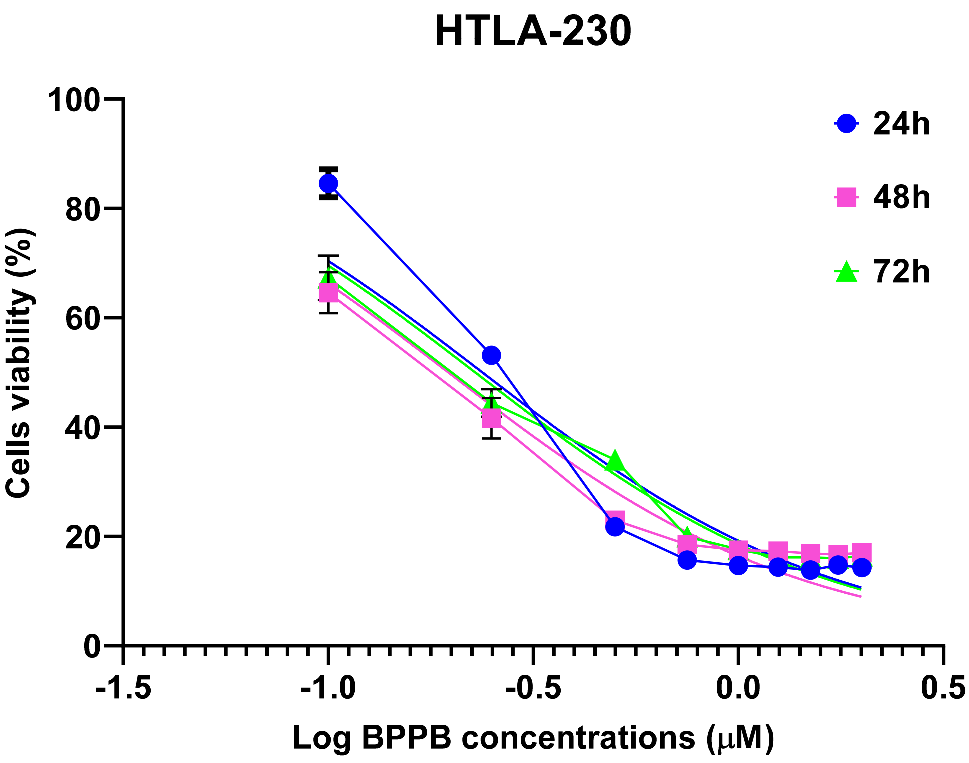

Figure S6. Plot of Log concentration of BPPB vs. cell viability (%) of HTLA-230 after 24, 48, and 72 hours of exposure (blue, pink and green traces with indicators and error bars) and plot of nonlinear fit of Log concentrations of BPPB vs. normalized response after 24, 48, and 72 hours of exposure (blue, pink and green traces without indicators).

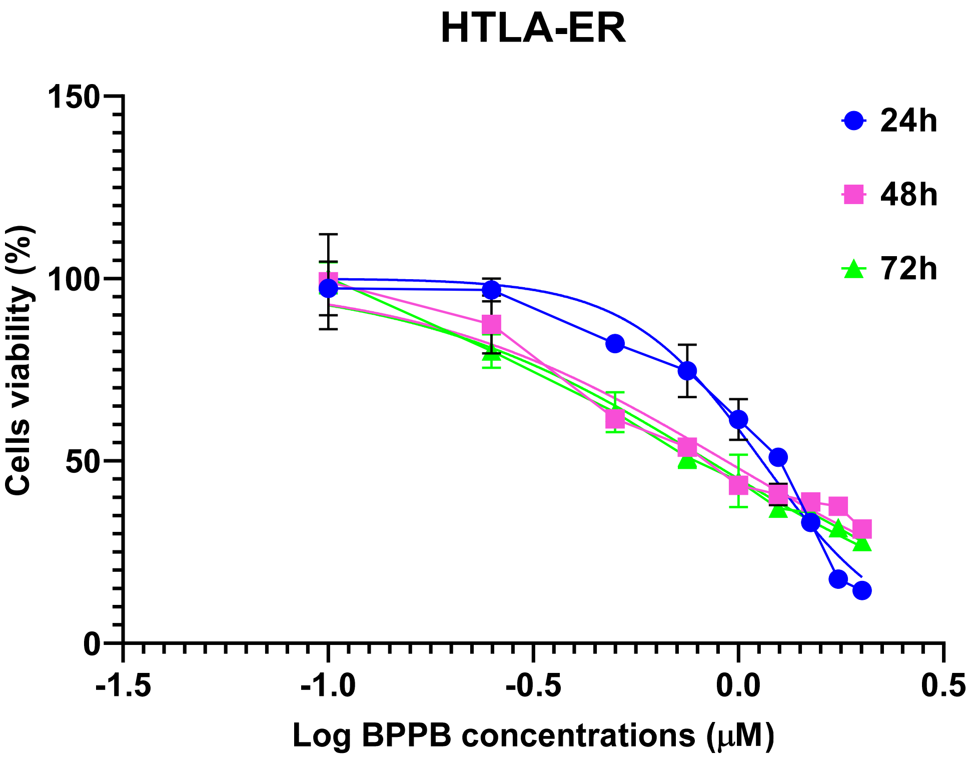

Figure S7. Plot of Log concentration of BPPB vs. cell viability (%) of HTLA-ER after 24, 48, and 72 hours of exposure (blue, pink and green traces with indicators and error bars) and plot of nonlinear fit of Log concentrations of BPPB vs. normalized response after 24, 48, and 72 hours of exposure (blue, pink and green traces without indicators).

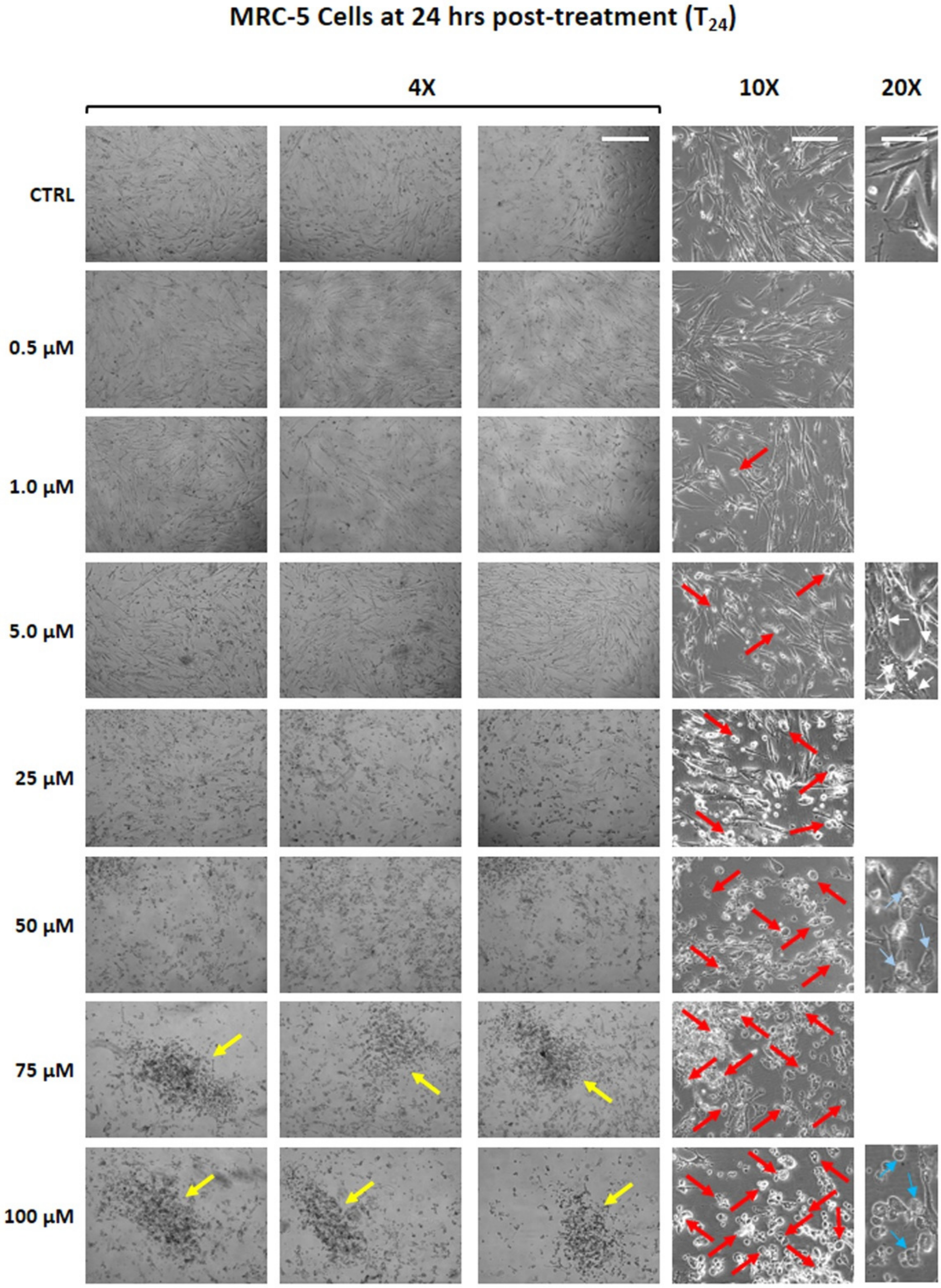

Figure S8. Bright filed images of triplicate wells (A, B and C as in Figure 6 in the main text) of MRC-5 cultured cells 24 hours post-treatment (T_{24}) at low (4 ×), medium (10 ×) and high (20 ×) enlargements. For 10 × and 20 × enlargements only one representative image is depicted. Yellow arrows: clusters of detached cells in suspension; red arrows: putative apoptotic cells. In 20 × images: white arrows: vacuoles; light blue arrows: rounded cells; blue arrows: detached cells. White dimensional bars correspond to 62.5, 25.0, 12.5 µm for 4 ×, 10 × and 20 × enlargements, respectively.

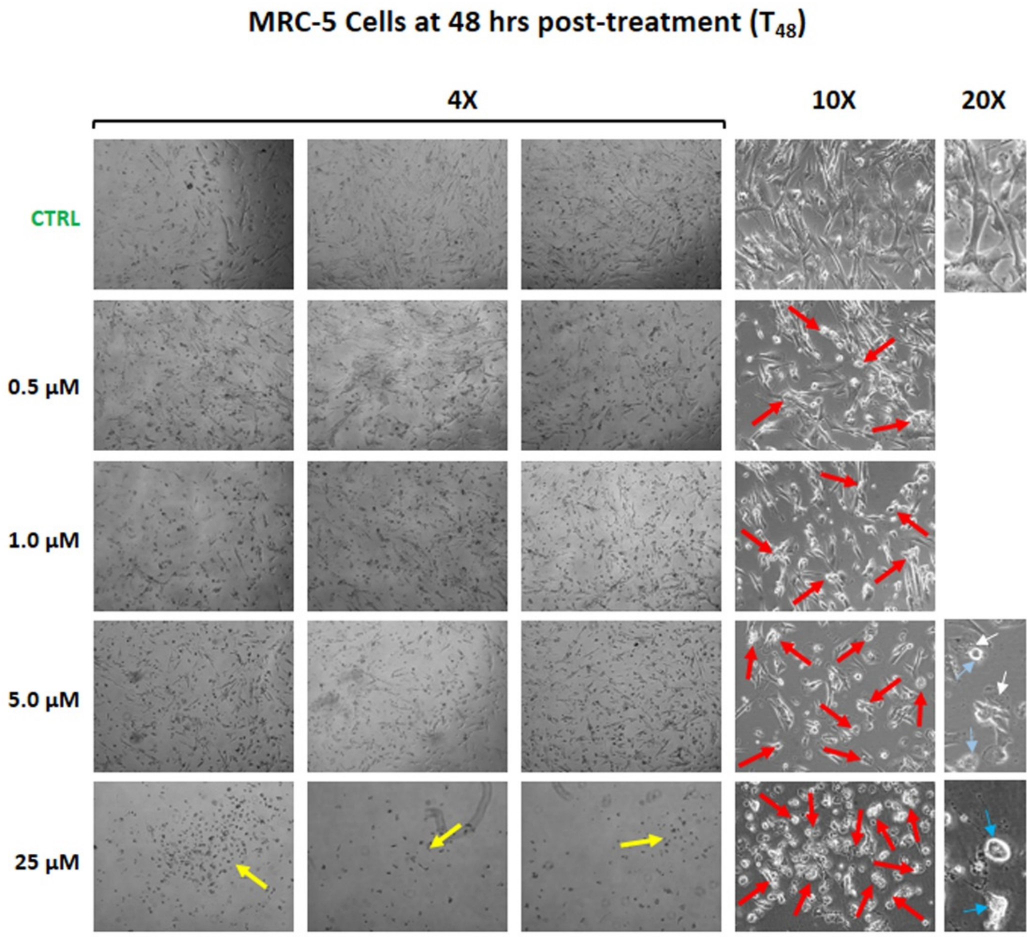

Figure S9. Bright filed images of triplicate wells (A, B and C, as in Figure 6 in the main text) of MRC-5 cultured cells 48 hours post-treatment (T_{48}) at low (4 ×), medium (10 ×) and high (20 ×) enlargements. Iconography as for Figure S8.

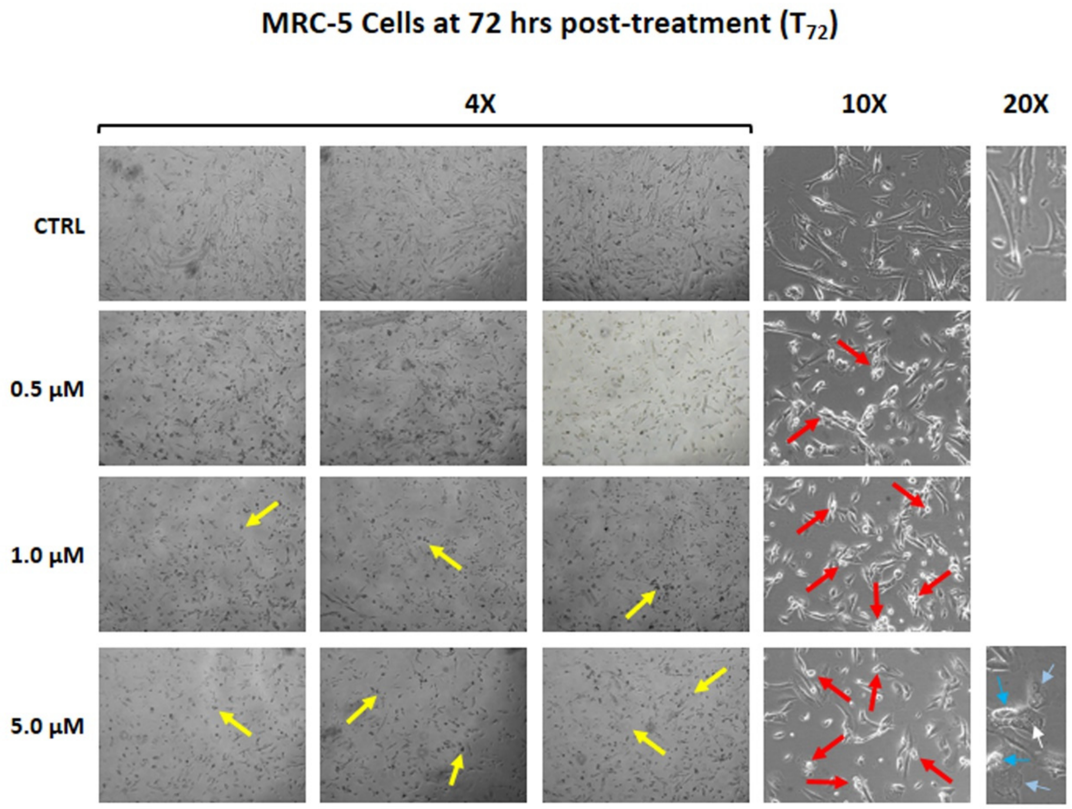

Figure S10. Bright filed images of triplicate wells (A, B and C, as in Figure 6 in the main text) of MRC-5 cultured cells 72 hours post-treatment (T_{72}) at low (4 ×), medium (10 ×) and high (20 ×) enlargements. Iconography as for Figure S8.

A

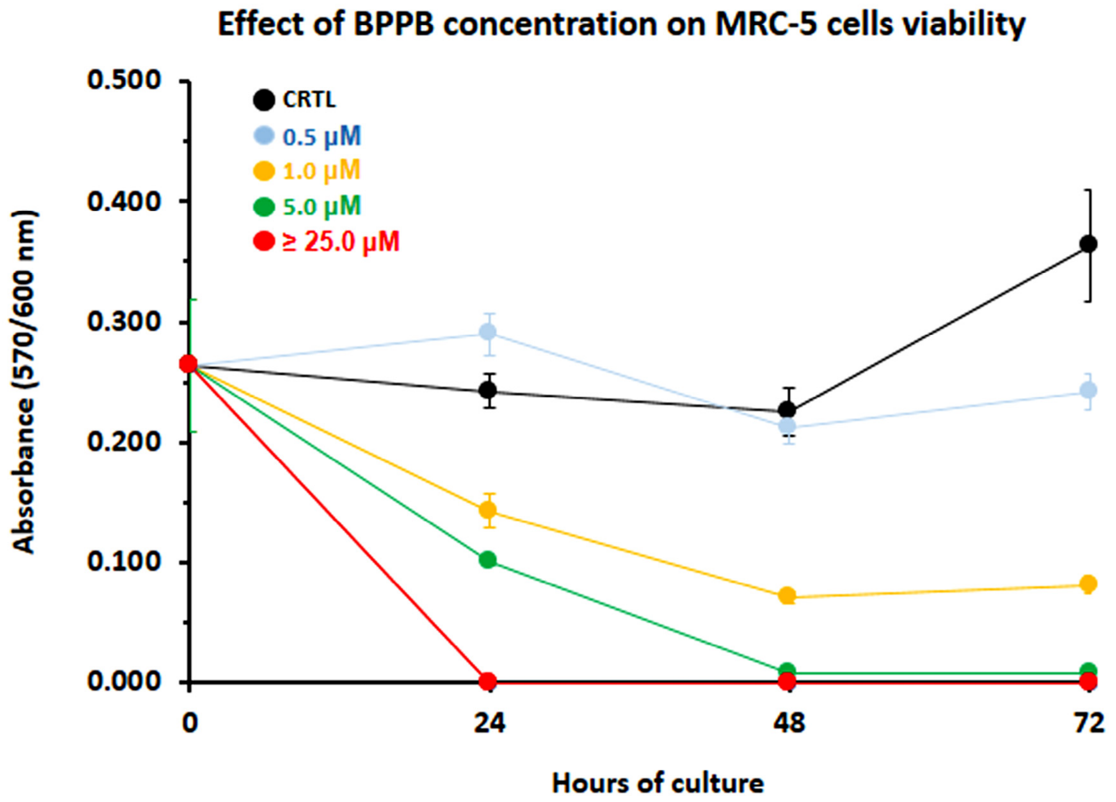

B

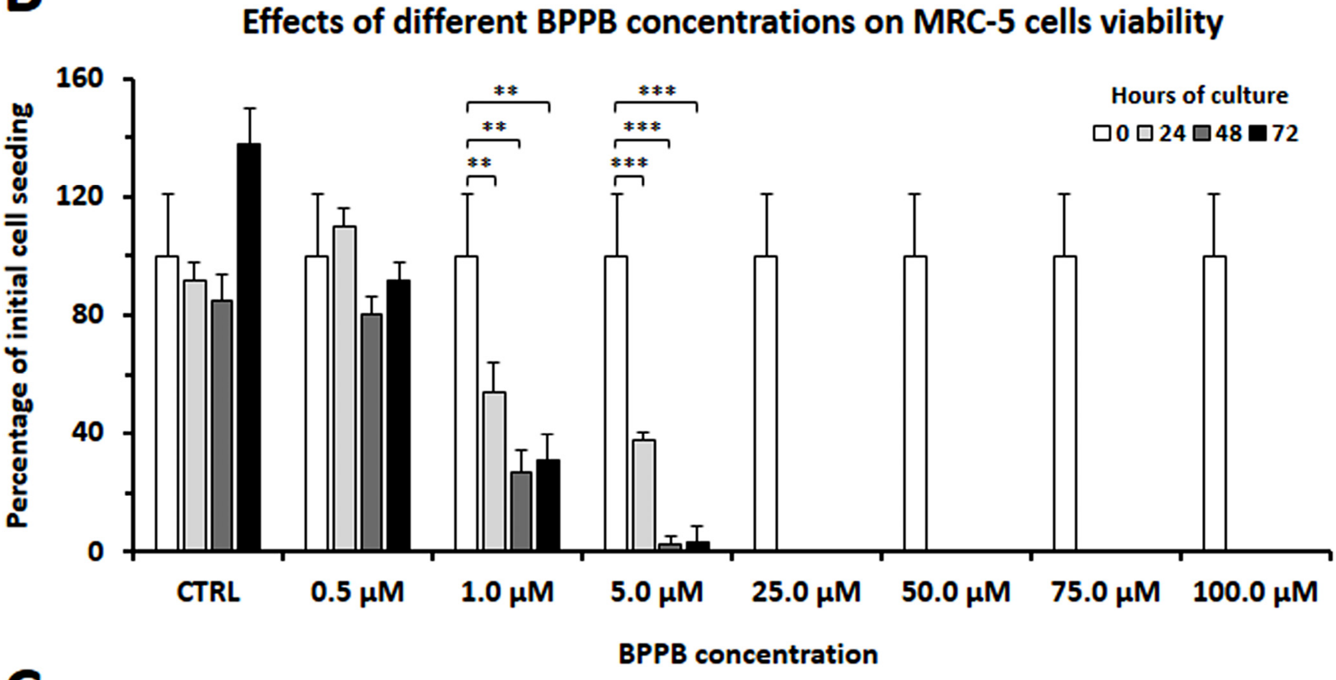

C

	BPPB concentration															
	CTRL		0.5 μM		1.0 μM		5.0 μM		25.0 μM		50.0 μM		75.0 μM		100.0 μM	
Hrs	%	SD%	%	SD%	%	SD%	%	SD%	%	SD%	%	SD%	%	SD%	%	SD%
0	100.00	20.82	100.00	20.82	100.00	20.82	100.00	20.82	100.00	20.82	100.00	20.82	100.00	20.82	100.00	20.82
24	91.92	5.93	109.89	6.05	54.05	9.83	38.01	2.21	0.00	0.00	0.00	0.00	0.00	0.00	0.00	0.00
48	85.30	8.70	80.32	6.14	26.81	7.57	2.78	2.68	0.00	0.00	0.00	0.00	0.00	0.00	0.00	0.00
72	137.64	12.64	91.49	6.19	30.63	9.33	3.04	5.78	0.00	0.00	0.00	0.00	0.00	0.00	0.00	0.00

Figure S11. Proliferation curves of MRC-5 cells upon different treatments and time points expressed as mean ± S.D. All curves referring to the highest BPPB concentrations (50.0, 75.0 or 100.0 μM; not shown) are superimposable to 25 μM data (red line). Error bars depict S.D. for each time point/concentration tested (N = 3, in duplicate) (A). Histogram depicts the average percentages of viable cells upon BPPB exposure at each concentration/time point tested, with respect to the initially seeded cells, ± S.D. For T_0, N = 24 (B). Table shows numerical data depicted in Figure S8B (C). Whenever indicated, **: 0.001 < p < 0.01; ***: p < 0.001 (Significance was determined by the single-factor ANOVA and Tukey post-hoc tests).

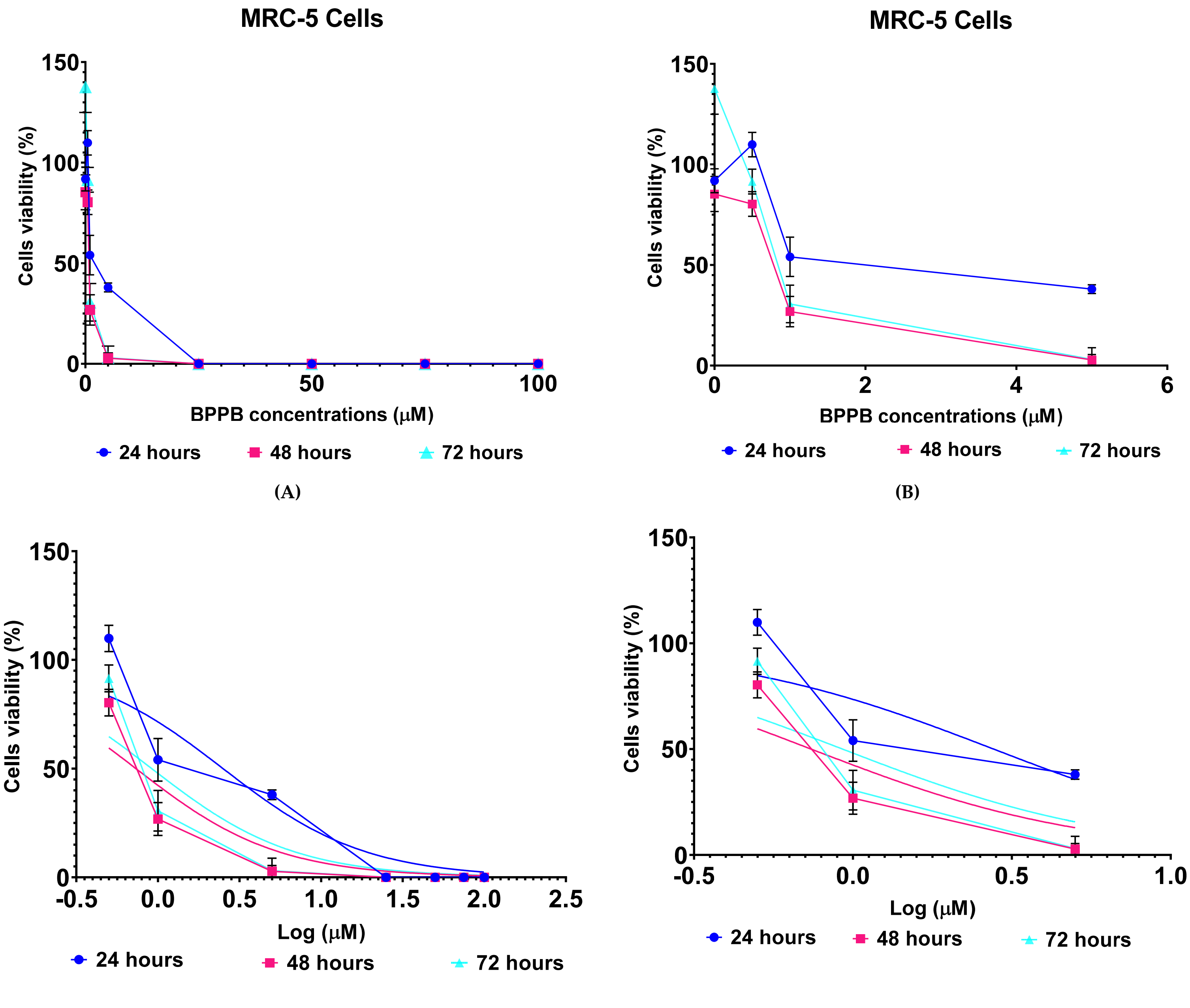

(C) (D)

Figure S12. Cell viability (%) of MRC-5 cells vs increasing BPPB concentrations (0.5-100 μM (A) and 0.5-5 μM (B)) after 24 hours (blue line) 48 hours (pink line) and 72 hours (light blue line) of exposure. Concentration = 0.0 μM corresponded to the controls.

Correspondent plots of Log concentration of BPPB (0.5-100 μM (C) and 0.5-5 μM (D)) vs. cell viability (%) of MRC-5 cells after 24, 48, and 72 hours of exposure (blue, pink and light blue traces with indicators and error bars) and plots of nonlinear fit of Log concentrations of BPPB vs. normalized response at 24, 48, and 72 hours of exposure (blue, pink and light blue traces without indicators).

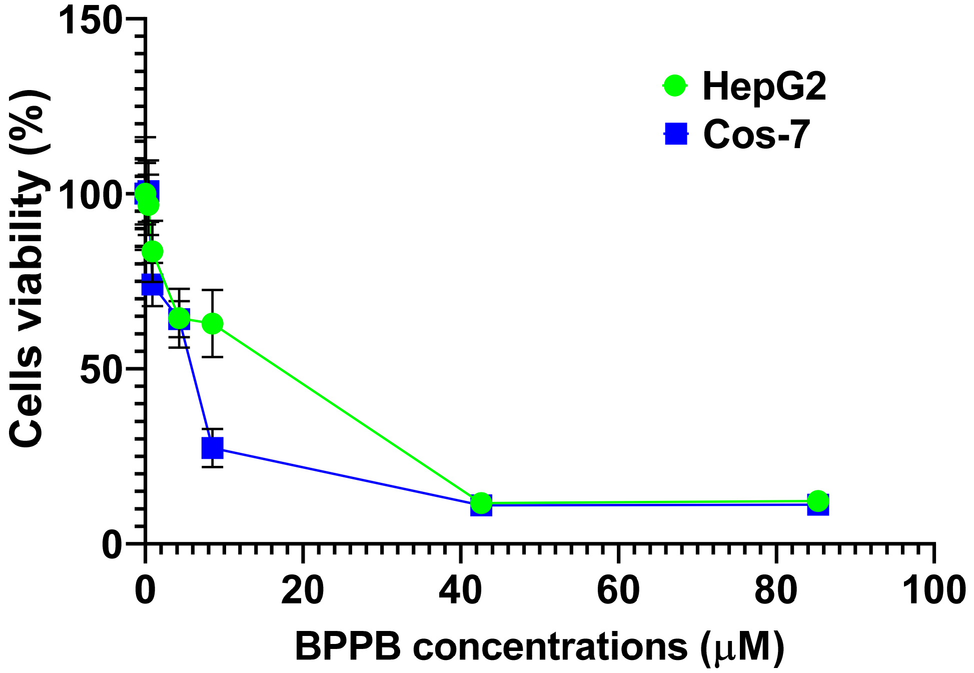

Figure S13. Cell viability (%) of Cos-7 (blue trace) and HepG2 (green trace) cells vs increasing BPPB concentrations (0.4-85.3 μM) after 24 hours of exposure. Concentration = 0.0 μM corresponded to the control.

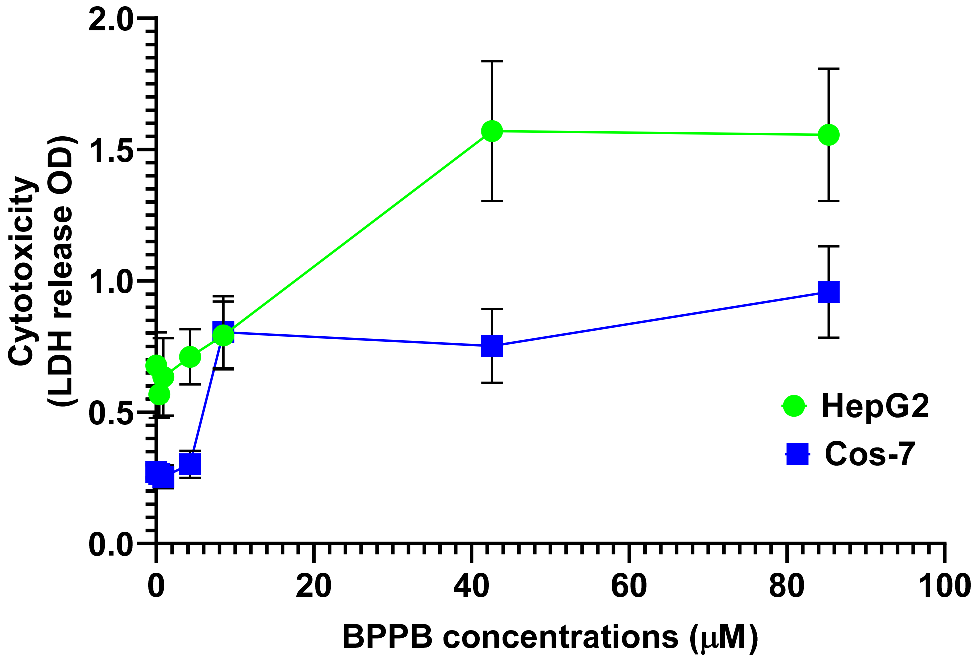

Figure S14. Cytotoxicity (LDH release OD) on Cos-7 (blue trace) and HepG2 (green trace) cells vs increasing BPPB concentrations (0.4-85.3 μM) after 24 hours of exposure. Concentration = 0.0 μM corresponded to the control.

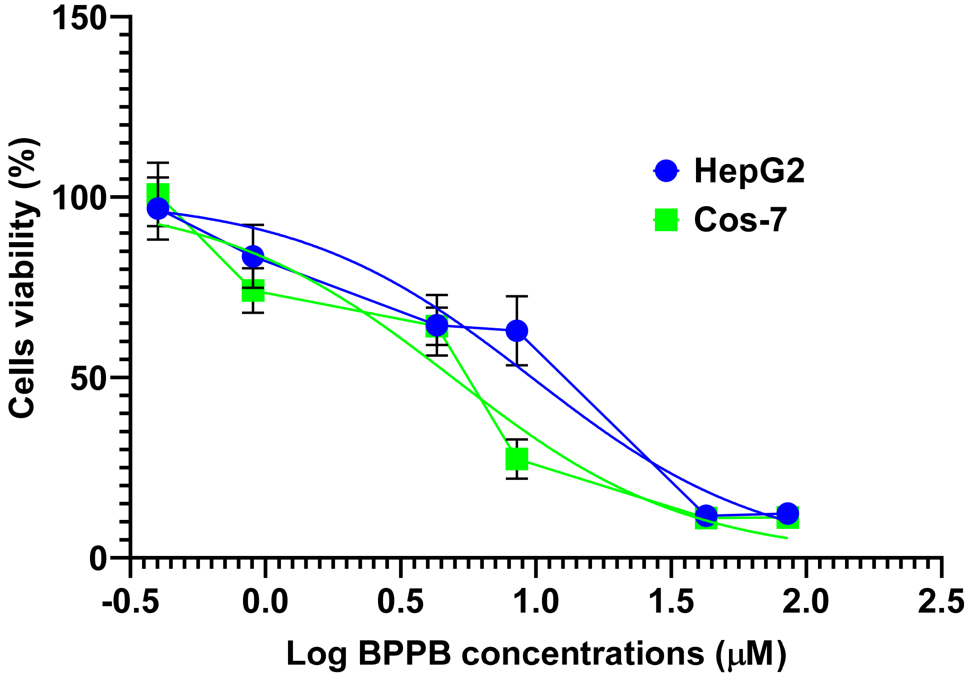

Figure S15. Plot of Log concentration of BPPB vs. cell viability (%) (blue and green traces with indicators and error bars) and plot of nonlinear fit of Log concentrations of BPPB vs. normalized response (blue and green traces without indicators).

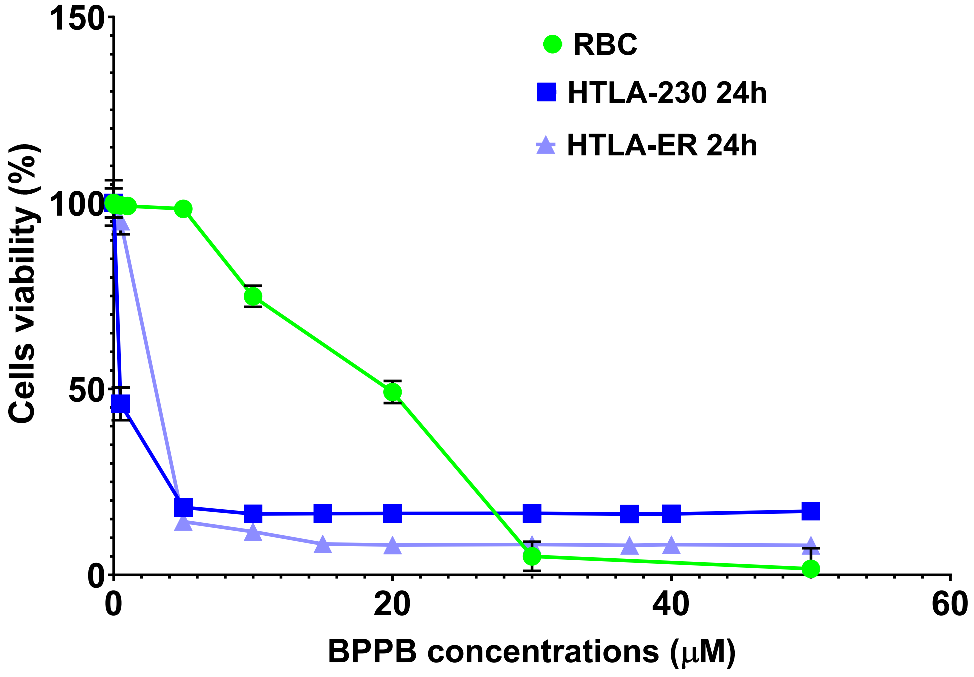

Figure S16. Cell viability (%) of RBCs (green trace), HTLA-230 (blue trace) and HTLA-ER cells (light blue trace) vs increasing BPPB concentrations (0.1-50 µM) after 24 hours (NB cells) or time of experiment of exposure. Concentration = 0.0 µM corresponded to the control.

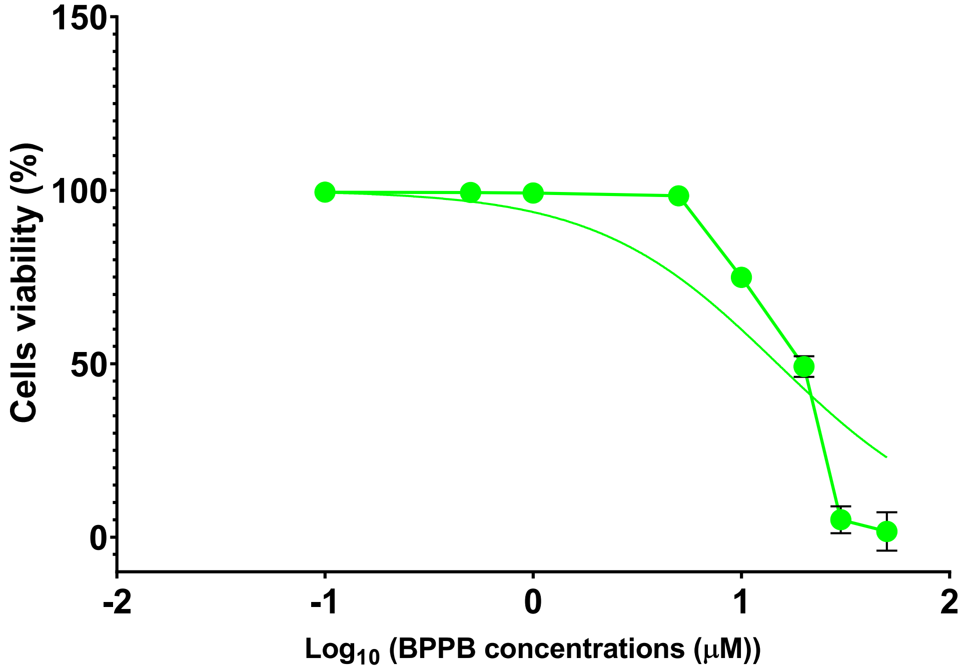

Figure S17. Plot of Log concentration of BPPB vs. RBCs viability (%) (green traces with indicators and error bars) and plot of nonlinear fit of Log concentrations of BPPB vs. normalized response (green traces without indicators).

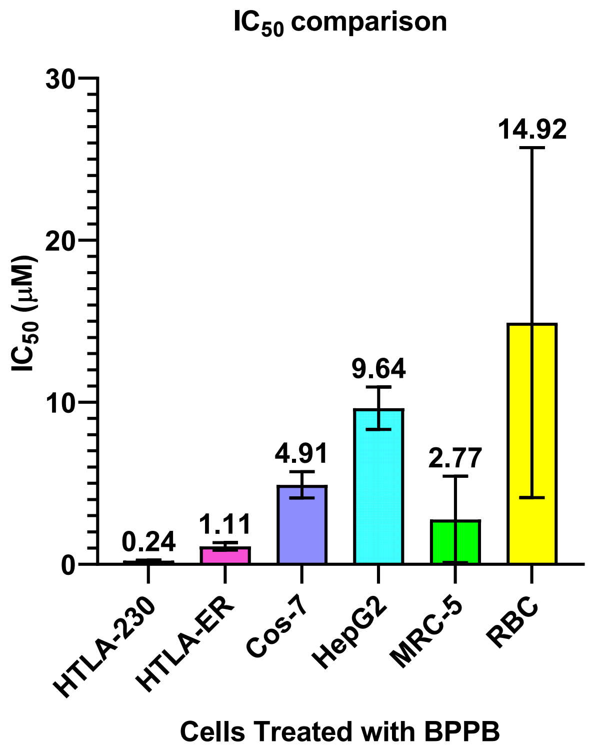

Figure S18. Bar graph comparing the IC_{50} values of BPPB on cancer cells (HTLA-230 and HTLA-ER) and normal ones (MRC-5, HepG2, Cos-7 and RBCs).
